# Second-Generation RT-QuIC Assay for the Diagnosis of Creutzfeldt-Jakob Disease Patients in Brazil

**DOI:** 10.3389/fbioe.2020.00929

**Published:** 2020-08-06

**Authors:** Breno José Alencar Pires Barbosa, Bruno Batitucci Castrillo, Ricardo Pires Alvim, Marcelo Houat de Brito, Helio R. Gomes, Sônia M. D. Brucki, Jerusa Smid, Ricardo Nitrini, Michele C. Landemberger, Vilma R. Martins, Jerson L. Silva, Tuane C. R. G. Vieira

**Affiliations:** ^1^Department of Neurology, Hospital das Clínicas, University of São Paulo Medical School, São Paulo, Brazil; ^2^Tumor Biology and Biomarkers Group, International Research Center, A.C. Camargo Cancer Center, São Paulo, Brazil; ^3^National Center of Nuclear Magnetic Resonance Jiri Jonas, Institute of Medical Biochemistry Leopoldo de Meis, National Institute of Science and Technology for Structural Biology and Bioimaging, Federal University of Rio de Janeiro-UFRJ, Rio de Janeiro, Brazil

**Keywords:** Creutzfeldt-Jakob disease, prion, rapidly progressive dementia, real-time quaking-induced conversion, biomarkers

## Abstract

The recent development of IQ-CSF, the second generation of real-time quaking-induced conversion (RT-QuIC) using cerebrospinal fluid (CSF), for the diagnosis of Creutzfeldt-Jakob Disease (CJD) represents a major diagnostic advance in the field. Highly accurate results have been reported with encouraging reproducibility among different centers. However, availability is still insufficient, and only a few research centers have access to the method in developing countries. In Brazil, we have had 603 suspected cases of CJD since 2005, when surveillance started. Of these, 404 were undiagnosed. This lack of diagnosis is due, among other factors, to the lack of a reference center for the diagnosis of these diseases in Brazil, resulting in some of these samples being sent abroad for analysis. The aim of this research study is to report the pilot use of IQ-CSF in a small cohort of Brazilian patients with possible or probable CJD, implementing a reference center in the country. We stored CSF samples from patients with possible, probable or genetic CJD (one case) during the time frame of December 2016 through June 2018. All CSF samples were processed according to standardized protocols without access to the clinical data. Eight patients presented to our team with rapidly progressive dementia and typical neurological signs of CJD. We used CSF samples from seven patients with other neurological conditions as negative controls. Five out of seven suspected cases had positive tests; two cases showed inconclusive results. Among controls, there was one false-positive (a CSF sample from a 5-year-old child with leukemia under treatment). The occurrence of a false positive in one of the negative control samples raises the possibility of the presence of interfering components in the CSF sample from patients with non-neurodegenerative pathologies. Our pilot results illustrate the feasibility of having CJD CSF samples tested in Brazilian centers and highlight the importance of interinstitutional collaboration to pursue a higher diagnostic accuracy in CJD in Brazil and Latin America.

## Introduction

Prion diseases, also known as transmissible spongiform encephalopathies (TSEs), encompass a group of rare neurodegenerative conditions secondary to abnormal conversion of a constitutively expressed cellular glycoprotein, the prion protein (PrP^C^), into an abnormally folded isoform (PrP^sc^) (Geschwind, 2016; Zanusso et al., 2016). Sporadic Creutzfeldt-Jakob disease (sCJD) is the most common prion disease in humans and usually presents as rapidly progressive dementia in combination with variable degrees of multisystem neurological impairment (Zerr and Hermann, 2018). Since its clinical and molecular manifestations are heterogeneous and non-specific, early diagnosis of prion diseases remains challenging in clinical practice (Geschwind and Murray, 2018).

Brain histopathological evaluation and/or detection of PrP^Sc^ are still the standard criteria for establishing a definitive diagnosis for CJD (CDC, 2018). However, invasiveness when performing this type of analysis *antemortem* brings very little benefit to the patient, since these diseases are still incurable. Clinical signs and paraclinical tests are the most commonly used approaches during the course of the disease and can be used to classify it as a possible or probable prion disease (Brown et al., 2003; CDC, 2018).

Among the paraclinical tests, brain diffusion weighted-MRI (DW-MRI) and cerebrospinal fluid (CSF) analysis have increased diagnostic accuracy (Eisenmenger et al., 2016; Staffaroni et al., 2019; Bizzi et al., 2020). The presence of 14-3-3 protein, Tau protein, neuron-specific enolase (NSE), the astroglial protein S100B and PrP^Sc^ itself are used as biomarkers in CFS for TSE diagnosis (Schmitz et al., 2016; Connor et al., 2019). Overall, the only protein that is a specific biomarker for TSE is PrP^Sc^. PrP^Sc^ can be detected in CSF based on its self-propagating ability: converting and seeding the aggregation of the non-pathogenic PrP^C^ into PrP^Sc^.

The real-time quaking-induced conversion (RT-QuIC) assay was developed in 2010, and since then, its experimental conditions have been improved and tested on a large number of samples in several laboratories worldwide (Wilham et al., 2010; Green, 2019). It ultrasensitively detects limited amounts of PrP^Sc^ in CSF and other tissue samples (Wilham et al., 2010). The recent development of the second-generation (IQ-CSF) RT-QuIC assay using CSF for the diagnosis of CJD represents a major diagnostic advance in the field (Wilham et al., 2010; Orrú et al., 2014). In humans, RT-QuIC analysis showed a sensitivity of 97.2% and specificity of 100%, with encouraging reproducibility among different centers (Franceschini et al., 2017).

RT-QuIC started to be clinically used in 2015 and became a criterion of the Centers for Disease Control and Prevention (CDC) to diagnose CJD as probable (CDC, 2018). Its high sensitivity and specificity make it an important clinical laboratory test for widespread use, but its global availability is still insufficient; only a few research centers have access to the method, especially in developing countries.

Surveillance of TSE cases has been compulsory in Brazil since 2005 (Martins et al., 2007), and 603 suspected CJD cases were reported up to June 2018. Of these, 404 were undiagnosed (de Oliveira Cardoso et al., 2015; Ministério da Saúde, 2018). Difficulties in performing imaging examinations and neuropathological analyses are found in several medical units in the country, making diagnosis problematic. Some diagnosed patients had samples sent to centers outside the country for biomarker analysis, allowing for greater coverage of the diagnostic criteria. The implementation of a specific test in Brazil, such as RT-QuIC, which can provide diagnosis for different TSEs with ease and confidence, is urgent to have notification and differential diagnosis for these diseases. This is also important to guide medical decisions. Here, we report the pilot use of the second-generation (IQ-CSF) RT-QuIC assay in a small cohort of Brazilian patients with possible or probable CJD to implement a reference center for this analysis in the country.

## Materials and Methods

### Clinical Investigation

**FIGURE 1:**
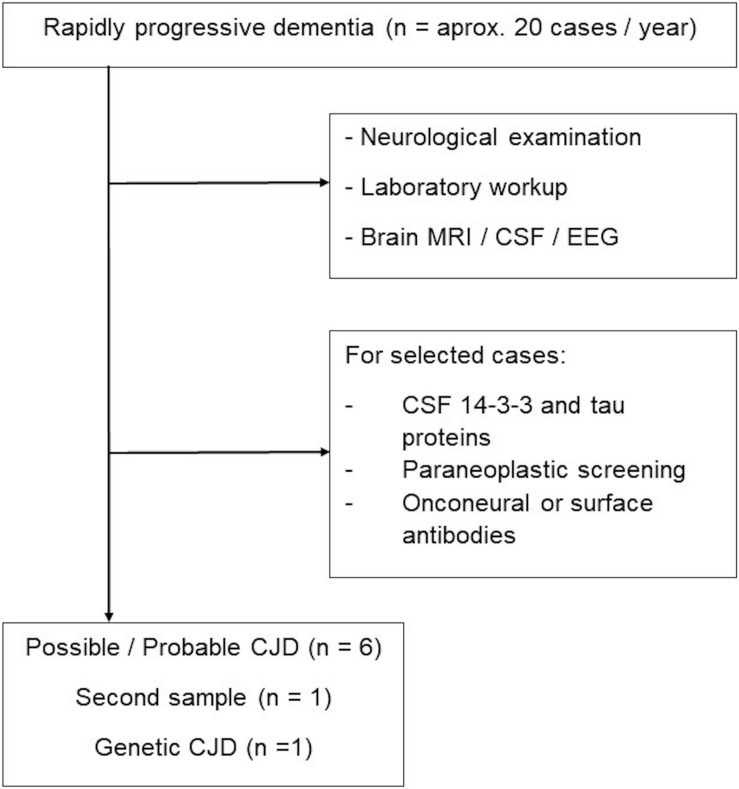
Flowchart of samples included for RT-QuIC analysis.

Patients with suspected CJD were admitted for investigation under a protocol for rapidly progressive dementia (RPD) as previously reported (Studart Neto et al., 2017). Following a full neurological examination, all patients underwent complete blood cell count, serum electrolytes, blood glucose, acute C-reactive protein, liver, renal and thyroid function tests, antithyroid and antinuclear antibody testing, as well as treponemal and HIV serology. Patients also underwent brain magnetic resonance imaging [T1, T2, fluid-attenuated inversion recovery (FLAIR), gradient echo, and diffusion weighted imaging sequences], EEG, and CSF (cell count, biochemistry, and g globulin levels). CSF 14-3-3 protein was assessed in cases of suspected prion diseases, as required by Brazilian norms. Selected patients underwent chest and abdomen computed tomography, mammography (for women), testicular ultrasound (for men), and thyroid ultrasound to rule out paraneoplastic RPD. In addition, we obtained onconeural and/or neuronal surface antigen antibody testing when paraneoplastic or autoimmune encephalitis was suspected. A flowchart for patient inclusion is provided in Figure 1.

Three cases had access to molecular analysis of the PRNP gene for polymorphisms in the 129 codon. Samples were analyzed using denaturing high-performance liquid chromatography. Technical details about this procedure as well as amplification reactions and DNA extraction have been previously described (Castro et al., 2004; Smid et al., 2013).

### CSF Samples

We analyzed eight CSF samples from seven patients with possible, probable or genetic CJD referred to the Department of Neurology at University of São Paulo from December 2016 to June 2018. One patient (named ABT) had her CSF tested twice at different times. We used CSF samples from seven patients with other neurological conditions as negative controls. CSF samples (10 mL) were collected by lumbar puncture (LP) following a standard procedure. Two milliliters of the CSF sample were then centrifuged at 1000 × g for 10 min and stored in polypropylene tubes at −80°C until blind analysis by researcher TCRGV at UFRJ.

### RT-QuIC

The RT-QuIC assay was performed using the improved QuIC-CSF (IQ-CSF) conditions as published (Orrú et al., 2015). Briefly, 20 μL of CSF was added to 80 μL of reaction mixture in each well of a black 96 well plate with a clear bottom (Nunc). The final solution contained 10 mM phosphate buffer at pH 7.4, 1 mM ethylenediaminetetraacetic acid tetrasodium salt dihydrate (EDTA) at pH 8.0, 300 mM NaCl, 10 μM thioflavin-T (ThT), 0.002% sodium dodecyl sulfate (SDS) and 0.1 mg/mL recombinant Syrian hamster truncated form of prion protein (Ha rPrP 90–231). Samples were tested in quadruplicate three or four times independently, generating a total of 12 or 16 wells evaluated for each sample. The plates were sealed and incubated in a FLUOstar OMEGA plate reader (BMG Labtech, Germany) at 55°C with intermittent cycles of shaking (60 s, double-orbital, 700 rpm) and rest (60 s). ThT fluorescence was collected every 45 min using 450 ± 10 nm (excitation) and 480 ± 10 nm (emission) wavelengths. The threshold was defined as the average fluorescence for all samples within the first 10 h of incubation plus 10 standard deviations (SD). A sample was considered positive when at least two of four replicate wells crossed this threshold. All IQ-CSF RT-QuIC analyses were performed at the Federal University of Rio de Janeiro.

Unseeded reaction (not shown) and random CSF samples from patients with other neurological conditions (Table 1) were selected as negative controls. Given the descriptive nature of this study, no statistical analyses were performed.

**TABLE 1:** Results of diagnostic investigations in the tested patient cohort.

**Sample, age**	**Diagnosis^*a*^**	**CSF analysis**					**RT-QuIC status**
		**WBC/μ L**	**Prot (mg/dL)**	**Glu (mg/dL)**	**Tau^*b*^ (pg/mL)**	**14-3-3**	
CT1, 55	Control (chronic meningitis)	23	192	30	–	–	Negative
CT2, 5	Control (leukemia)	2	22	45	–	–	Positive
CT3, 62	Control (cranial nerves syndrome)	1	71	102	–	–	Negative
HCS, 63	Control (multiple sclerosis)	1	43	80	–	–	Negative
HBV, 41	Control (HIV)	3	35	66	–	–	Negative
RMC, 64	Control (SAH/ventriculitis)	25	134	54	–	–	Negative
ABS, 43	Control (chronic meningitis)	9	62	40	–	–	Negative
LRC, 69	RPD (probable CJD)	1	27	61	2279	Negative	Positive^*d*^
DAS, 69	RDP (probable CJD)	5	32	63	2109	Positive	Positive^*d*^
ABT1, 74	RPD (possible CJD)	1^c^	39	71	908	Negative	Negative
ABT2, 74	RPD (possible CJD)	1	32	61	NA	NA	Positive
ASM, NA	RPD (genetic CJD)	NA					Positive
JJN, 65	RPD (probable CJD)	1	66	61	519	NA	Positive
GMT, 75	RPD (possible CJD)	4	97	61	115	NA	Negative
TN, 73	RPD (probable CJD)	1	79	96	NA	Negative	Positive

**WBC, white blood cells; Prot, protein levels; Glu, glucose levels; RPD, rapidly progressive dementia; SAH, subarachnoid hemorrhage; NA, not available. ^*a*^Controls and their suspected diagnosis/investigation. ^*b*^Reference value for total tau levels was <450 pg/mL. ^*c*^This sample had 33 red blood cells. ^*d*^These patients also had their CSF samples sent abroad with positive results (The National Prion Disease Pathology Surveillance Center, Cleveland, OH, United States).**

## Results

In the referred time frame, seven patients presented to our team with rapidly progressive dementia and typical neurological signs of CJD. The patient named ABT had her CSF tested twice at different times (ABT1 and ABT2 samples), therefore rendering a total of eight CSF samples. ABT patient results will be better reported in Case 1 description.

**FIGURE 2:**
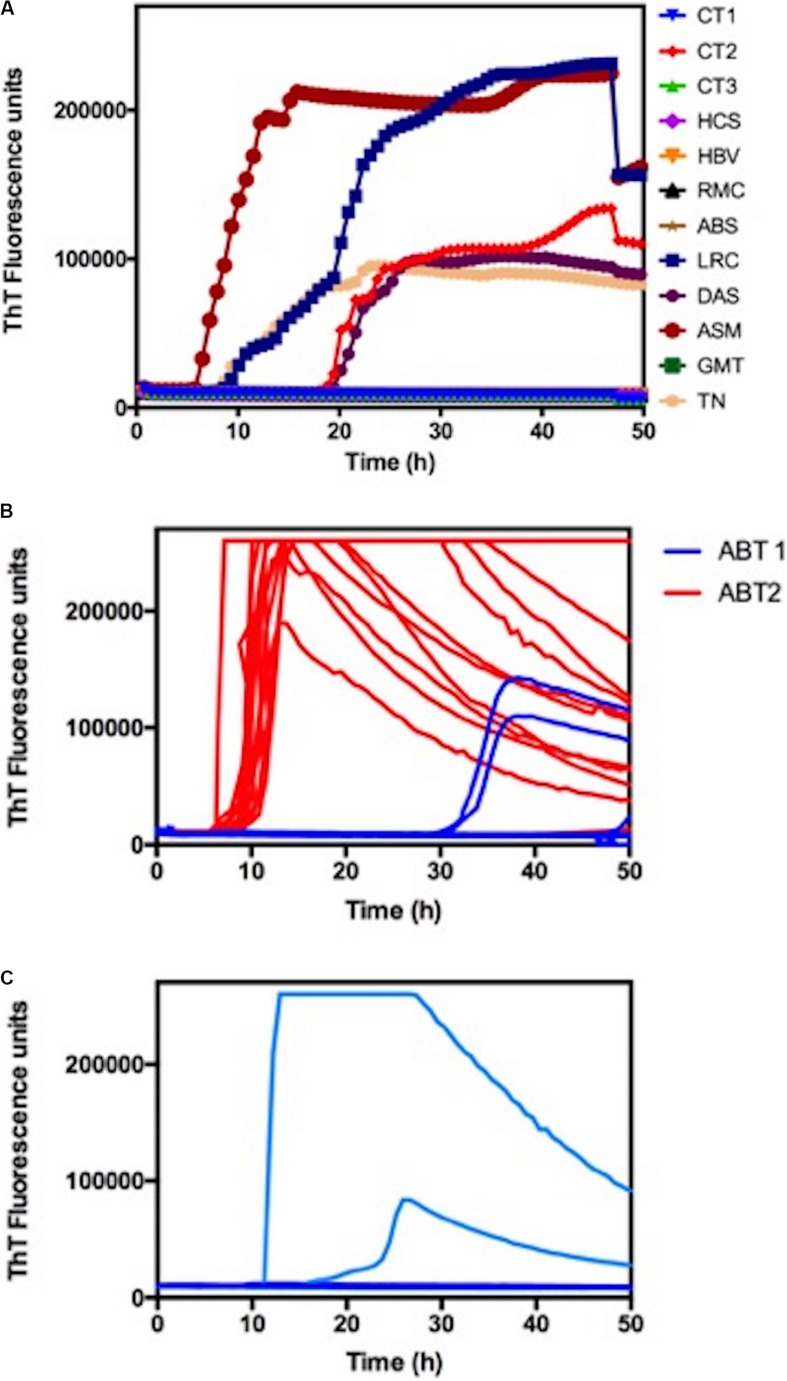
Blind RT-QuIC analysis of CSF samples. **(A)** RT-QuIC responses from reactions seeded with 20 μl of CSF from suspected CJD cases (LRC; DAS; ASM; JJN; GMT, TN). The RT-QuIC responses from reactions seeded with CSF samples from patients with other neurological disorders (CT1; CT2; CT3; HCS; HBV; RMC; ABS) were used as a negative control. Each curve represents the mean of four replicate readings of three or four repetitions. **(B)** Single-well analysis of CSF samples from patient ABT. ABT1 and ABT2 refer to the first and second collections, respectively. **(C)** Single-well analysis of CSF samples from patient JJN.

We randomly used CSF samples from seven patients with other neurological conditions as negative controls. Five out of eight suspected samples had positive RT-QuIC results in our hands (Figure 2A). Clinical diagnosis, CSF analysis and RT-QuIC results are summarized in Table 1.

Samples from patients LRC and DAS were used as positive controls, given that they were previously analyzed using RT-QuIC in a reference center outside the country (The National Prion Disease Pathology Surveillance Center, Cleveland, OH, United States). Our RT-QuIC analyses were also positive, corroborating this result (Figure 2A). Patient ASM was a genetic CJD positive for the E200K mutation, and in our analysis, this patient was also positive, presenting a very short lag phase and a high fluorescence signal (Figure 2A).

Among the negative controls, there was one false positive, sample CT2 (Figure 2A). The false-positive case was a 5-year-old child with leukemia who was receiving intrathecal chemotherapy (GBTLI protocol with methotrexate, aracitin, citrabin, dexamethasone) during the time frame of the study.

In the following, two cases are presented in which the results obtained with RT-QuIC show interesting aspects related to RT-QuIC sensitivity and disease progression.

### Case 1

**FIGURE 3:**
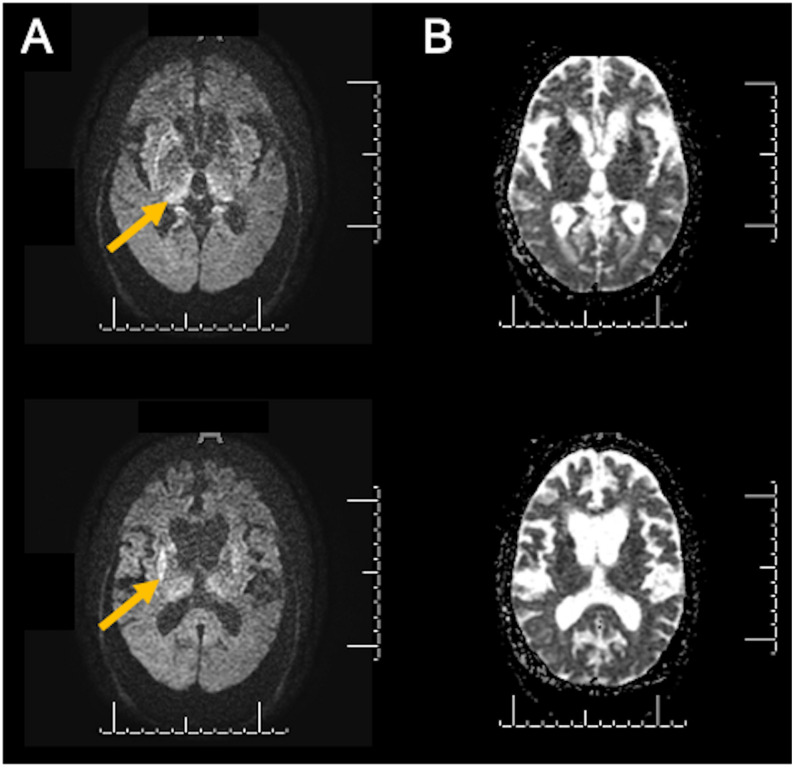
Diffusion-weighted images (DWI) from ABT (case 1) revealing enlarged ventricles and symmetric hyperintensities in the basal ganglia (**A**, yellow arrows). **(B)** shows corresponding apparent diffusion coefficient (ADC) hypointensities in the same territory.

ABT was a 72-year-old woman with 7 years of formal education. She had an atypical presentation characterized by a 4-month history of rapidly progressive cognitive impairment associated with visual hallucinations and gait disturbances with repeated falls. She had no relevant past medical history or family history of any neurological conditions. On neurological examination, she scored 17/30 on the Mini-Mental State Examination (MMSE), and physical tests revealed only a prominent axial syndrome as she could not sit or stand up without bilateral support. There were no pyramidal signs, parkinsonism or visuospatial impairment. A comprehensive investigation with metabolic, inflammatory, paraneoplastic, and infectious tests was unremarkable. Brain magnetic resonance imaging (MRI) revealed symmetric diffusion weighted image (DWI) hyperintensities in the basal ganglia (Figure 3), a finding that raised the suspicion of CDJ. The CSF analysis was unremarkable with elevated tau protein levels and negative 14-3-3. The RT-QuIC results were inconclusive; once from 16 wells, only two crossed the threshold (ABT1 sample in Figure 2B). Electroencephalogram (EEG) revealed disorganized electrical brain activity with no periodic waves. Three months later, she developed a significant worsening, with the need for aid for most activities of daily living, severe cognitive impairment (she could not be tested with the MMSE), myoclonus and the presence of prominent frontal reflexes on neurological examination. At this point, a prolonged EEG eventually showed bilateral periodic sharp waves, and another lumbar puncture was performed with a second sample sent for RT-QuIC analysis, with a positive result with a high fluorescence (ABT2 sample in Figure 2B). She eventually died of clinical complications 11 months after the first symptoms appeared. PRNP gene analysis revealed codon 129 heterozygous MV.

### Case 2

**FIGURE 4:**
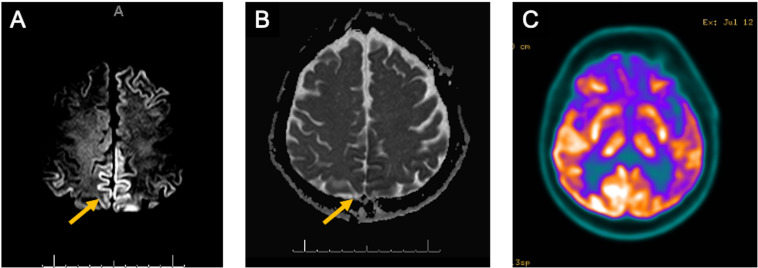
**(A)** MRI diffusion-weighted images from JJN (case 2) revealing hyperintensities on frontal, temporoparietal and posterior cingulate cortical areas (**A**, yellow arrow); **(B)** MRI apparent diffusion coefficient maps with corresponding hypointensities in the same regions above (**B**, yellow arrow); **(C)** 18-FDG PET/CT shows hypometabolism on the bilateral temporoparietal cortex, posterior cingulate, precuneus, and caudate nucleus.

JJN was a 65-year-old male who presented to our clinic with an 8-month history of behavioral changes. He had 4 years of education and a medical history of hypertension and gouty arthritis. There was no family history of any neurological conditions. His wife described the first symptoms as prominent changes in his food preferences with an unusual demand for rice and chicken. Two months later, he developed visual hallucinations, mostly described as the presence of spiders in the ceiling. In the first evaluation, he was independent of instrumental activities of daily living. Neurological examination revealed an MMSE of 20/30 with an unremarkable physical examination. A laboratory work-up including metabolic, inflammatory and serology studies was negative. At this point, we were surprised by the finding of brain MRI diffusion weighted imaging revealing marked bilateral hypersignal in the frontotemporoparieto-occipital cortex, basal ganglia, thalamus and – less markedly – in the hippocampus, raising the suspicion of sporadic Creutzfeldt-Jakob Disease (sCJD) (Figures 4A,B). The patient was hospitalized for further investigation. The EEG study revealed bilateral and synchronous slow waves. A brain 18-FDG PET showed hypometabolism in the temporal and frontal lobes, caudate nuclei and temporoparietal cortex (Figure 4C). The CSF study was initially unremarkable, except for the high total tau protein levels, whereas 14-3-3, p-tau and beta-amyloid values were within the normal range. A CSF sample was sent for autoantibodies and RT-QuIC testing. The patient was empirically treated with intravenous methylprednisolone for 5 days and was discharged for outpatient follow-up. At this point, we received CSF results negative for autoantibodies with an inconclusive RT-QuIC response; from 12 wells, only two crossed the threshold (Figure 2C). In the following months, he eventually developed cognitive deterioration and parkinsonism, with the presence of more complex visual hallucinations. He eventually died of clinical complications approximately 28 months after initial symptoms. PRNP gene analysis revealed codon 129 heterozygous MV.

## Discussion

The present study reports the results of pilot second-generation RT-QuIC testing in a small patient cohort referred for rapidly progressive neurological syndromes of suspected prion nature. Our center previously reported 61 cases of rapidly progressive dementia among 1648 patients in a 3-year interval. Immune-mediated encephalopathies represented the majority of the samples (46%), followed by CJD (11.5%) (Studart Neto et al., 2017). All cases from that study were diagnosed with CJD according to the University of California San Francisco Modified Criteria (Vitali et al., 2011), which does not include CSF testing.

As we described here, IQ-CSF RT-QuIC proved to be an extremely important tool in the diagnosis of ABT and JJN cases (Cases 1 and 2). Both patients presented with rapidly progressive neurological syndromes and could not initially be classified with possible or probable CJD, according to the most recent criteria (CDC, 2018). Both ABT and JJN showed slightly elevated levels of total Tau protein, but although this biomarker has 87–90% sensitivity, it is only 67–75% specific for CJD (Connor et al., 2019). 14-3-3 protein was not elevated in both cases, despite their genetic subtype MV, which is often associated with positive 14-3-3 protein levels (Manix et al., 2015).

In the case of ABT patient, the clinical presentation of rapid cognitive impairment with visual hallucinations and gait impairment was considered atypical. In this case, the brain MRI was an important diagnostic clue, with DWI hyperintensities in the basal ganglia leading to a differential diagnosis with metabolic encephalopathies, hypoxic-ischemic lesions or toxic lesions (i.e., carbon monoxide intoxication) (Finelli and DiMario, 2003). A careful clinical assessment made all possibilities less likely, and CJD became our main hypothesis. Despite an inconclusive RT-QuIC result for the first collected sample, the patient eventually evolved with a more typical impairment, and the second CSF sample with a 3-month interval yielded positive RT-QuIC.

The different results obtained with the ABT patient samples were very intriguing. We hypothesize that this difference could be attributed to higher loads of PrP^sc^ following disease progression. Although disease duration does not seem to be related to RT-QuIC results (McGuire et al., 2012), it is not yet clear whether there is a change in the presence of seeds in CSF according to disease progression. To our knowledge, this approach performed with samples of a patient collected at different times has not yet been tested, and this is the first report suggesting that RT-QuIC results change according to disease evolution. A more feasible hypothesis would be that the first sample had some interference, such as blood that could mask the positive result (Cramm et al., 2016), despite a only modest presence of red blood cells in this particular sample (33 cells/μL, see Table 1).

Atypical features for sCJD in JJN (Case 2) included (1) a presentation with mild behavioral changes; (2) the initial sparing of motor systems, with evidence of motor findings in the neurological examination only almost 12 months after first symptoms; (3) an unremarkable EEG with 7–8 months of evolution; and (4) the presence of a bilateral T2 hypersignal in the hippocampus. The RT-QuIC was inconclusive, but together with MRI, it could suggest early diagnosis of probable CJD. Perhaps with disease progression, JJN’s CSF sample would obtain a positive RT-QuIC result, as observed for ABT patient.

The occurrence of one false-positive case in our RT-QuIC test weakens our diagnostic accuracy and underscores the need for improvements in the protocol. Despite having an extremely high specificity, IQ-CSF RT-QuIC false-positive results have been reported in the literature. Hayashi et al. (2017) reported the case of a 61-year-old man who presented with rapid cognitive impairment, myoclonus and recurrent seizures. A brain MRI revealed cortical hyperintensities, and the CSF analysis showed elevated 14-3-3 and tau levels, with a positive RT-QuIC. Despite aggressive treatment with corticotherapy, the patient died, and post-mortem assessment revealed only pathological changes after convulsion, with no signs of prion disease. The authors conclude that convulsion may cause false-positive RT-QUIC results and that a post-mortem evaluation remains the gold standard for diagnosing similar cases. The shaking effect was analyzed *in vitro* by Orrú et al. (2016), and they showed that long shaking periods reduced scrapie-seeded reaction times, but continuous shaking promoted false-positive reactions.

Regarding our false-positive case, we did not find any similar cases reporting the use of intrathecal medications as a possible reason for a positive RT-QuIC result. A positive case in a 5-year-old child without any neurological symptoms would never be expected; an 18-year-old woman was the youngest person diagnosed with probable sCJD using RT-QuIC (Yao et al., 2017). Therefore, this sample was selected as a negative control, although infant CSF was never RT-QuIC analyzed. Despite optimized protocols, sample processing issues can always be a possibility for such unexpected results.

The present study is limited by its pilot nature, with a modest sample size. Improvements in the establishment of the protocol are necessary, requiring a greater number of analyses. The use of nasal brushes to collect patient samples for RT-QuIC analysis is also valid to improve protocol sensitivity and specificity. However, this is the first study to our knowledge to report specific biomarker-based feasible results performed in a developing country for prion diagnosis, in addition to pointing out new possible interferences in the protocol, and the need to understand how the different current diagnostic approaches can reveal disease progression.

## Conclusion

The cases reported here illustrate the importance of using RT-QuIC for patients with neurological syndromes, enabling the diagnosis of probable CJD, while no other method was sufficient to support this diagnosis, even with atypical clinical presentation. In addition, the identification of a false positive in a sample from a leukemic pediatric patient undergoing intrathecal treatment with chemotherapy suggests new possible interferences in the method. This will require future investigation of the effect of these chemotherapeutic agents for inclusion or not as a limitation for carrying out the assay.

CSF samples are often evaluated only once, due to the invasiveness of CSF collection and the absence of curative treatment for TSEs, in addition to rapid disease progression. One of the cases we report points out the importance of carrying out studies that evaluate the progression of the disease and that RT-QuIC is a useful approach for such studies. In this case, the use of nasal brushes might be prioritized over CSF analysis, since this provides greater sensitivity and enables more frequent sample collection, given the less invasive nature of the procedure.

Finally, our study illustrates the feasibility of having CJD CSF samples tested in Brazilian centers, and highlights the importance of inter-institutional collaboration in order to pursue a greater diagnostic accuracy for CJD in developing countries. It also demonstrates that RT-QuIC can be clinically available for testing patients from Brazil and other Latin American countries, and points to the need and feasibility of establishing a national reference center for the diagnosis of these rare but devastating diseases.

## Data Availability Statement

All datasets generated for this study are included in the article/supplementary material.

## Ethics Statement

Ethical review and approval was not required for the study on human participants in accordance with the local legislation and institutional requirements. Written informed consent to participate in this study was provided by the participants’ legal guardian/next of kin. Written informed consent was obtained from the individual(s), and minor(s)’ legal guardian/next of kin, for the publication of any potentially identifiable images or data included in this article.

## Author Contributions

BB: study design, patient data collection, and manuscript writing. BB, BC, RA, MB, HG, and SB: patient data collection and interpretation. JS and RN: study design, data interpretation, and manuscript critical revision. ML and VM: genetic analysis. JLS and TV: RT-QuIC facility implementation. TV: study design, RT-QuIC evaluations, RT-QuIC data analysis, and manuscript writing. All authors contributed to the article and approved the submitted version.

## Conflict of Interest

The authors declare that the research was conducted in the absence of any commercial or financial relationships that could be construed as a potential conflict of interest.
